# When Nostalgia Tilts to Sad: Anticipatory and Personal Nostalgia

**DOI:** 10.3389/fpsyg.2020.01186

**Published:** 2020-05-29

**Authors:** Krystine I. Batcho

**Affiliations:** Le Moyne College, Syracuse, NY, United States

**Keywords:** personal nostalgia, anticipatory nostalgia, stories, personality, emotion

## Abstract

Contemporary research has showcased many benefits of nostalgia, but its bittersweet character and historical reputation as unhealthy raise the possibility of less favorable impacts. In recent studies, daily diary data highlighted nostalgia’s mixed valence and suggested that nostalgia is more strongly associated with negative feelings. Variables that influence the adaptive or maladaptive dimensions of nostalgia have not yet been fully explored. Recently, a focus on *when* nostalgia is experienced relative to past and future was introduced in the construct of anticipatory nostalgia, missing the present prematurely before it has become past. Distinct from personal nostalgia, anticipatory nostalgia was found to be characterized by difficulty enjoying the present and a tendency toward sadness and worry. The present study examines the distinctive dynamics at play in anticipatory and personal nostalgia by exploring the relationship between each type of dispositional nostalgia and reported experience with happy and sad stories. The Nostalgia Inventory, the Survey of Anticipatory Nostalgia, and a brief form of the PANAS were completed by 144 undergraduates (110 women), who rated their exposure and reactions to happy and sad stories. Reported frequency of exposure to happy and sad stories was related to dispositional happiness and sadness. Personal and anticipatory nostalgia did not differ in frequency of exposure to happy and sad stories, but they did differ in reactivity to and learning from sad stories. Findings highlight the importance of the timing of nostalgia, consistent with the distinction between nostalgia for the past and nostalgia for what is still present.

## Introduction

In contrast to nostalgia’s dark origins in medical history as a disease construct, contemporary researchers have largely rediscovered nostalgia as a healthy psychological phenomenon. Empirical evidence has contradicted historical arguments that nostalgia reflects an inability to accept the loss of the past or serves as an obstacle to living in the present or moving forward (Werman, 1977; Kaplan, 1984, 1987). Presumed maladaptive qualities have been replaced by documented beneficial functions, including strengthened social connectedness, continuity of self, enhanced self-esteem, adaptive coping strategies, meaning in life, and comfort in the face of threatened mortality (Batcho, 1998, 2013; Wildschut et al., 2006, 2010; Batcho et al., 2008; Routledge et al., 2008; Zhou et al., 2008; Iyer and Jetten, 2011; Cheung et al., 2013; Stephan et al., 2014; Sedikides et al., 2015).

However, recent research has revived questions about possible unfavorable aspects of nostalgia by highlighting its distinctive bittersweet nature (Larsen et al., 2001; Larsen and McGraw, 2011, 2014; Hepper et al., 2012). While most studies have found that nostalgia increases positive affect, instances of elevated negative affect, including sadness, have been identified (Zinchenko, 2011; Stephan et al., 2012; Newman et al., 2019). The extent to which nostalgia is uplifting and helpful or bitter and counterproductive might depend upon personality traits or contextual variables (Barrett et al., 2010; Köneke, 2010; Wildschut et al., 2010, 2019; Hart et al., 2011; Iyer and Jetten, 2011; Seehusen et al., 2013; Abeyta et al., 2014; Garrido, 2018). One largely unexplored variable is the timing of nostalgia (Batcho, 2020). As an experience that unfolds over time, nostalgia’s impact might depend upon the cognitive appraisal that directs the feelings and thoughts generated during a nostalgic episode.

When nostalgia focuses on loss that has not yet occurred, the sadness of anticipated loss is premature and the experience becomes a paradoxical phenomenon of enjoying the present while missing it as if already relegated to the past. Dubbed anticipatory nostalgia, such premature nostalgia has been introduced as a construct in its own right (Batcho and Shikh, 2016; Bergs et al., 2019). A form of nostalgia by definition, it can be distinguished from personal nostalgia. Personal nostalgia is missing what has been lost, whereas anticipatory nostalgia involves missing what has not yet been lost. Anticipatory nostalgia depends upon mentally creating an imagined future that gives rise to missing what will be “someday past,” yet still present. By engaging abstract construal, anticipatory nostalgia might engender psychological distance from the present, decreasing direct involvement in the current concrete reality (Nussbaum et al., 2003).

Anticipatory nostalgia should not be confused with *anticipated nostalgia*. Anticipated nostalgia is the prediction or expectation that one will feel nostalgic for an aspect of the present *in the future*, not feeling nostalgic in the present. Predicting future nostalgia is an interesting cognitive process but not identical to the emotional phenomenon of feeling nostalgic before the future loss occurs. A person can expect to miss a loved one when they leave or die someday, but the expectation doesn’t necessarily include the emotional component of nostalgic missing. Expecting future nostalgia has been shown to predict nostalgia after an important life transition. The finding of a greater likelihood of expecting future nostalgia for more positive experiences is not surprising, as people would be likely to expect to miss enjoyable or valued events. Expecting future nostalgia was associated with greater savoring of the experience, and post-transition nostalgia was associated with benefits, including enhanced self-esteem, social connectedness, and meaning in life (Cheung et al., 2019).

By contrast, it is not clear whether *anticipatory nostalgia*, premature missing of what is still present, shares the benefits of nostalgia for the past. Despite eliciting the sadness of loss, anticipatory nostalgia might also allow reappraisal of the present. In spite of or because of the sadness, anticipating loss during adversity might encourage effective coping by strengthening appreciation of what is good in the present and providing the comfort of knowing that difficulties will not last. Initial empirical investigations to identify the benefits or disadvantages of anticipatory nostalgia assessed anticipatory nostalgia as a dispositional trait, that is, as the tendency of individuals to experience it generally. The early studies supported the viability of anticipatory nostalgia as a construct distinguishable from personal nostalgia (Batcho and Shikh, 2016). Unlike dispositional personal nostalgia, dispositional anticipatory nostalgia was characterized by a greater tendency for people and experiences to cause worry and sadness and was more likely to occur in adverse circumstances.

The present study extends the early research by exploring the relationship between nostalgia and experience with stories. A substantial amount of the existing research on nostalgia has examined memories. Like memories, stories entail the past inherently by recounting what has happened in the past. Stories differ from memories in that the content of stories does not necessarily originate from an individual’s own experience. Empirical research exploring stories is voluminous and well-established, but has focused primarily on identifying features of stories, context, and cognitive processing that influence retention and emotional and behavioral impacts (Habermas and Diel, 2010; Dunlop et al., 2011; McGinty et al., 2013; Berger et al., 2019; Landrum et al., 2019). Not yet adequately explored is the enduring or cumulative influence of emotional stories, especially those that convey personal meaning. Qualitative analyses have shown that personally relevant stories can exert a powerful lasting influence (Pratt and Fiese, 2004; Kiser et al., 2010). In their memoirs, members of the resistance during World War II explained how stories encountered during childhood generated nostalgia and played pivotal roles in their dedication to the resistance, even to the point of enduring hardships and risking death (Batcho, 2018).

The present work introduced a focus on accumulated exposure to stories over time. In an investigation of the relationship between overall exposure to stories and personal and anticipatory nostalgia, participants estimated and shared their experience with happy and sad stories. If experiencing nostalgia prematurely is less adaptive than missing what is already past, anticipatory nostalgia will be aligned with unfavorable reactions to stories, whereas personal nostalgia will be associated with positive reactions. In particular, exposure to happy and sad stories was examined to explore whether dispositional anticipatory nostalgia is associated with greater exposure, reactivity, or attraction to sad stories. If the association of anticipatory nostalgia with sadness reflects an overall sad disposition, anticipatory nostalgia would be expected to correlate with greater exposure to or recall of sad stories. If anticipatory nostalgia develops in response to sad experiences, reported exposure to sad stories would be expected to correlate with anticipatory nostalgia. However, prior research has not examined the possibility of cognitive benefits. If anticipating future loss entails helpful cognitive processing, anticipatory nostalgia will be associated with greater likelihood of learning from sad stories.

## Methods

### Participants

A sample of 144 undergraduates, 110 women and 34 men, ranging in age from 18 to 39 years (*Mdn* = 20, *SD* = 2.65), completed the study. In accordance with ethical norms of the American Psychological Association, participants were offered either a small stipend or optional extra credit for a course for their participation. The offer of a stipend helped recruit students majoring in different disciplines.

### Material and Procedure

All procedures and material for the study were reviewed and approved by the college Institutional Review Board for compliance with ethical guidelines. In small groups in a laboratory room, participants completed paper forms of the Nostalgia Inventory, the Survey of Anticipatory Nostalgia, selected items from Watson and Clark’s (1994) Positive and Negative Affect Schedule-Expanded Form (PANAS-X), and a survey of experience with happy and sad stories constructed for this study.

The Nostalgia Inventory assessed personal nostalgia as a dispositional trait (Batcho, 1995, 1998, 2007). Consistent with Stern’s (1992) definition of nostalgia as the longing for one’s past, respondents rate the extent to which they miss each of 20 items from when they were younger on a 9-point scale (1 = *Not at all*, 9 = *Very much*). The inventory is reported to have a split-half reliability of 0.78 and 1-week test-retest reliability of 0.84 (Batcho, 1995), an acceptable level of internal consistency of 0.86 as measured by Cronbach’s alpha (Batcho et al., 2008), and test-retest reliability of 0.82 over a 4-week interval (Batcho et al., 2011). Consistent with prior research, the Nostalgia Inventory yielded an acceptable level of internal consistency of 0.88 as measured by Cronbach’s alpha and a split-half reliability of 0.81 in this study.

The Survey of Anticipatory Nostalgia focuses on future loss rather than the past (e.g., “society will change”). Given a 9-point scale (1 = *rarely/not very*, 9 = *very often/very much*), respondents were instructed: “Sometimes we realize that something won’t last forever. It can be an activity, a thing, or time with a person or group. Using the scale below, CIRCLE a number to estimate how often or to what extent you feel like you already miss each of the items before the change has happened.” The survey is reported to have a split-half reliability of 0.83, 4-week test-retest reliability of 0.71, and internal consistency as measured by Cronbach’s alpha of 0.87 (Batcho and Shikh, 2016). Consistent with prior research, internal consistency as measured by Cronbach’s alpha was 0.88, and split-half reliability measured 0.79 in this study.

As in prior research (Batcho and Shikh, 2016), mean ratings on the Nostalgia Inventory correlated moderately with mean ratings on the Survey of Anticipatory Nostalgia, *r*_(__142__)_ = 0.52, *p* < 0.001.

The Survey of Experience with Stories (Appendix) included eight items asking participants to rate their experience with stories on a 9-point scale (1 = *rarely/not at all*, 9 = *very often/very much*). Participants rated how often they had been exposed to happy and to sad stories in general, how happy and how sad happy and sad stories made them feel, how often they choose happy and sad stories, and how often they benefit or learn from happy and sad stories. Participants were asked to describe the earliest happy and sad story they remember, as well as additional happy and sad stories. They were instructed: “You might have read it or it might have been told to you, read to you, depicted in a video or film, or have been a news story. You might have thought it was a true story or a fictional one.”

Consistent with prior research (Batcho and Shikh, 2016), six items were selected (*cheerful*, *happy*, *joyful, sad*, *blue*, and *downhearted*) from the PANAS-X (Watson and Clark, 1994). Given the dispositional or trait instruction, participants rated the extent to which they had felt each item “in general, that is, on the average” on a 5-point scale (1 = *very slightly or not at all*, 5 = *extremely*).

## Results

### Gender

Men and women did not differ in average personal nostalgia scores, *t*(1, 142) = −1.768, *p* = 0.084, η*_*p*_*^2^ = 0.029, but women (*M* = 6.00, *SD* = 1.18) scored higher in anticipatory nostalgia than did men (*M* = 5.24, *SD* = 1.20), *t*(1, 142) = −3.29, *p* = 0.001, η*_*p*_*^2^ = 0.071.

### Nostalgia and Positive and Negative Affect

Ratings of PANAS-X items of generalized affect yielded additional evidence for anticipatory nostalgia as a distinct construct. Ratings from the three items *cheerful*, *happy*, and *joyful* were averaged to yield a composite measure of a tendency toward happiness. Ratings from the three items *sad*, *blue*, and *downhearted* were averaged to serve as a measure of sadness. Personal nostalgia was not correlated with happiness, *r*_(__142__)_ = 0.06, *p* = 0.476, or sadness, *r*_(__142__)_ = 0.12, *p* = 0.136, whereas anticipatory nostalgia correlated significantly with sadness, *r*_(__142__)_ = 0.21, *p* = 0.012, but not with happiness, *r*_(__142__)_ = 0.14, *p* = 0.087.

### Stories

#### Nostalgia and Experience With Happy and Sad Stories

The relationship of personal and anticipatory nostalgia to exposure and reactions to happy and sad stories was explored in correlational analyses, with gender and dispositional sadness and happiness controlled (Table 1). Frequency of exposure to happy and sad stories was assessed in two ways. Participants were asked to rate overall exposure by rating how often they encountered happy and sad stories. They also recalled happy and sad stories in open-ended essays, and the number of stories reported served as an additional measure of exposure. Participants were free to recall as many stories as they could without a time limit. Subjective estimates and recall entail different cognitive processes. Consistent with such differences, the ratings and number of stories recalled were not correlated for happy stories, *r*_(__142__)_ = 0.01, *p* = 0.922, or sad stories, *r*_(__142__)_ = 0.11, *p* = 0.197, and served as independent measures. Neither type of nostalgia correlated significantly with either indicator of frequency of exposure to happy and sad stories, suggesting that people prone to personal or anticipatory nostalgia have not experienced happy and sad stories more or less often. Attraction to or preference for sad or happy stories was assessed by asking participants to rate how often they choose happy and sad stories. Neither type of nostalgia correlated significantly with story choice, suggesting that proneness to personal or anticipatory nostalgia does not reflect an underlying bias or preference for happy or sad material.

**TABLE 1:** Partial correlations of personal and anticipatory nostalgia with exposure and reactions to stories with gender and dispositional happiness and sadness controlled.

	**Personal** **nostalgia**		**Anticipatory** **nostalgia**	
	***r***	***p***	***r***	***p***
Number of happy stories	–0.04	0.634	0.03	0.743
Number of sad stories	0.02	0.829	0.06	0.466
**Ratings**				
Exposure to happy stories	0.03	0.693	0.02	0.777
Exposure to sad stories	0.11	0.209	0.06	0.452
Choice of happy stories	0.13	0.118	0.09	0.303
Choice of sad stories	0.05	0.565	0.14	0.102
Happy stories make happy	0.19	0.026	0.03	0.694
Sad stories make sad	0.23	0.007	0.21	0.012
Learn from happy stories	0.16	0.052	0.09	0.294
Learn from sad stories	0.05	0.584	0.21	0.012

*N = 142. Controlled for gender and dispositional sadness and happiness measured by PANAS-X. PANAS-X happiness composite included Cheerful, Joyful, Happy. Sadness composite included Sad, Blue, Downhearted.*

However, ratings suggested that they react differently to happy and sad stories emotionally and cognitively. Emotionally, personal and anticipatory nostalgia both correlated with sadness induced by sad stories, but only personal nostalgia correlated significantly with happiness elicited by happy stories. Results suggested that personal and anticipatory nostalgia differed in cognitive engagement with stories. Personal nostalgia correlated with learning from happy stories, whereas anticipatory nostalgia correlated with learning from sad stories. Consistent with prior findings of an association between anticipatory nostalgia and a tendency to be made sad by people and events, these results suggested that people prone to anticipatory nostalgia may be less reactive to happy stories. Present findings suggested that people prone to anticipatory nostalgia process sad stories in more productive ways, whereas people prone to personal nostalgia are more reactive to happy stories and process happy stories in beneficial ways.

#### Contributions of Nostalgia and Stories to Mood

The contributions of nostalgia and stories to dispositional mood were explored further in linear regression analyses (Table 2). The composite happiness ratings were examined in a linear regression analysis, with gender and mean ratings from the Nostalgia Inventory and the Survey of Anticipatory Nostalgia independent variables. The role of stories was explored with the inclusion of mean ratings of frequency of exposure to happy and sad stories, degree of happiness (sadness) induced by happy (sad) stories, and learning from happy and sad stories.

**TABLE 2:** Summary of regression analyses on dispositional mood.

**Dispositional mood**	**B**	**SE**	**β**	***t***	***p***
**Dispositional happiness**					
Gender	–0.01	0.13	–0.01	–0.03	0.980
Personal nostalgia	–0.04	0.05	–0.08	–0.89	0.375
Anticipatory nostalgia	0.06	0.06	0.10	1.03	0.306
Exposure to happy stories	0.10	0.04	0.23	2.75	0.007
Exposure to sad stories	0.04	0.03	0.09	1.13	0.261
Happiness elicited by happy stories	0.19	0.05	0.36	3.82	0.000
Sadness elicited by sad stories	–0.04	0.04	–0.10	–1.20	0.232
Learning from happy stories	0.02	0.04	0.04	0.44	0.660
Learning from sad stories	0.02	0.03	0.04	0.52	0.604
**Dispositional sadness**					
Gender	0.10	0.18	0.05	0.56	0.580
Personal nostalgia	–0.01	0.07	–0.02	–0.20	0.844
Anticipatory nostalgia	0.11	0.07	0.15	1.47	0.145
Exposure to happy stories	–0.04	0.05	–0.08	–0.87	0.386
Exposure to sad stories	0.10	0.04	0.21	2.40	0.018
Happiness elicited by happy stories	0.03	0.07	0.05	0.52	0.602
Sadness elicited by sad stories	0.08	0.05	0.15	1.60	0.113
Learning from happy stories	0.01	0.05	0.01	0.14	0.890
Learning from sad stories	0.01	0.04	0.01	0.04	0.970

*N = 143.*

In a significant model, *F*(9, 133) = 5.53, *p* < 0.001, Adjusted *R*^2^ = 0.223, gender, personal nostalgia, and anticipatory nostalgia were not significant. Dispositional happiness was predicted by the frequency of exposure to happy, but not sad stories. Similarly, happiness was predicted by the degree to which happy stories induce happiness, but not by the degree to which sad stories induce sadness, consistent with an affect-specific effect. The absence of significant effects of learning from happy or sad stories was consistent with a distinction between affective and cognitive impacts.

A parallel analysis was conducted with the composite sadness ratings as the dependent variable. In a significant model, *F*(9, 133) = 1.92, *p* = 0.05, Adjusted *R*^2^ = 0.055, gender, personal nostalgia, and anticipatory nostalgia were not significant. Dispositional sadness was predicted by the frequency of exposure to sad, but not happy stories. However, sadness was not predicted by the sadness elicited by sad stories, happiness induced by happy stories, or learning from sad or happy stories.

#### Limitations

This study introduced a focus on stories as a vehicle for comparing emotional and cognitive reactions to cumulative meaningful experiences associated with personal and anticipatory nostalgia. Not allowing causal relationships to be determined, this exploratory study encourages future research based on experimental designs to identify variables responsible for the differences between personal and anticipatory nostalgia. Further research is needed also to explore participant variables in samples characterized by broader demographic and diversity constituents.

A fuller understanding of adaptive functions of nostalgia would be advanced with studies that identify the nature and content of the stories participants prone to personal or anticipatory nostalgia were exposed to and remember. Similarly, future work is needed to determine how those prone to nostalgia learn or benefit from happy or sad stories. Qualitative analyses of the impactful stories can yield insights into the nature of the lessons acquired.

## Discussion

The present findings reinforced the importance of when nostalgia is felt relative to past and future. Results are consistent with the view of anticipatory nostalgia as distinguishable from personal nostalgia and worthy of further research as a distinct phenomenon. Stories were shown to be effective material for elucidating the roles of emotional and cognitive processes in personal and anticipatory nostalgia. Exploring reactions to stories can clarify the adaptive and maladaptive functions of nostalgia within a meaningful practical context. Access to 24 h news cycles and online venues have expanded the influence of the content and format of stories. The current findings highlight the importance of future research to understand the impact of immersion in a story-rich environment.

The present study suggests the possibility of a cumulative contribution of experience with stories to emotional well-being. Greater exposure to happy stories correlated with higher levels of dispositional happiness, and greater exposure to sad stories correlated with higher levels of dispositional sadness. The overall pattern of relationships is interesting. While dispositional happiness was related to the degree to which stories elicited happiness, sadness was related to experience with sad stories regardless of the degree of sadness the stories elicited. The present findings suggest that the emotional impact of a story depends in part upon the emotional and cognitive reactivity of the listener or viewer.

Pertinent to the current study, personal and anticipatory nostalgia were distinguished by different ways of interacting with stories. There was no evidence that differential exposure to happy and sad stories accounted for the distinction. Personal and anticipatory nostalgia both correlated with being made sad by sad stories, suggesting that participants prone to anticipatory nostalgia are not more sensitive to sad stories. However, only personal nostalgia was associated with being made happy by happy stories, suggesting that anticipatory nostalgia may be associated with less reactivity to happy stories. Furthermore, personal nostalgia was related to greater likelihood of learning from happy stories, whereas anticipatory nostalgia was associated with greater likelihood of learning from sad stories.

Prior research differentiated personal and anticipatory nostalgia by their relationships to happiness and sadness. Personal and anticipatory nostalgia both include missing, longing or yearning. In personal nostalgia, we enjoy valued aspects of our past again, thereby enjoying them twice. In anticipatory nostalgia, we have only the present to enjoy them. In fact, we feel the sadness of missing them twice, once in the actual present and once in the imagined future. Envisioning what the future might bring can be accompanied by sadness missing the present and worry about what will come next. In prior research, participants prone to anticipatory nostalgia had reported a greater tendency for people and experiences to cause them worry and sadness, but anticipatory nostalgia did not predict generalized sadness. Consistent with prior research, anticipatory nostalgia did not contribute to generalized sadness in this study, suggesting that people prone to anticipatory nostalgia are not generally unhappy.

Given that personal and anticipatory nostalgia are both emotional constructs, theorists are justified in comparing their emotional facets. The present findings highlight the wisdom of considering also the cognitive dynamics in the relationship of nostalgia to the impact of stories. Rather than generally unhappy, people prone to anticipatory nostalgia may be more likely to benefit from sad stories by learning ways of coping with current or future problems and anxiety. Their inclination to imagine the future as present may reflect conceptual thinking that enables them to apply lessons from stories to their own lives. It remains for future investigations to identify the variables or conditions underlying the emotional and cognitive dynamics that distinguish personal from anticipatory nostalgia.

The present findings encourage further research to determine the long-term effects of being immersed in happy or sad stories. The relationship of learning from sad stories with anticipatory nostalgia suggests that benefiting from stories may be facilitated by an ability to imagine how the survival or resolution of sadness in a story might apply to an individual’s own life. Just as the sadness of future loss can be experienced before it is actualized, in adverse times, imagining when difficulties will have been overcome can be a source of consolation. But looking ahead brings the sad realization that the good of the present might be gone too. Future research is needed to determine if anticipatory nostalgia can be constructive in certain circumstances or applied therapeutically to remind people that time and the opportunity to be engaged in the present is fleeting. Sad stories may provide opportunities to deal with adversity safely from a distance, as anticipatory nostalgia allows loss to be confronted from a temporal distance.

In a digital age, children are growing up with expanding opportunities for exposure to stories with negative content presented in graphic media without the comforting presence of adults who can contribute meaningful perspective. The cumulative impact over time is not yet known, but the present findings suggest greater dispositional sadness with increasing exposure to sad stories. Current findings highlight the importance of identifying ways of cultivating traits or cognitive-emotional resources to buffer adverse outcomes. While sadness might be considered a maladaptive affective dimension of anticipatory nostalgia, the benefits of learning from sad material may suggest there are untapped adaptive cognitive functions of anticipatory nostalgia.

## Data Availability Statement

The datasets for this article are not publicly available because participants were not so instructed. Requests to access the datasets should be directed to KB, batcho@lemoyne.edu.

## Ethics Statement

The studies involving human participants were reviewed and approved by Le Moyne College Institutional Review Board. The patients/participants provided their written informed consent to participate in this study.

## Author Contributions

The author confirms being the sole contributor of this work and has approved it for publication.

## Conflict of Interest

The authors declare that the research was conducted in the absence of any commercial or financial relationships that could be construed as a potential conflict of interest.

## Appendix

### Survey of Experience With Stories

In general, how often have you been exposed to **happy** stories?

In general, how often have you been exposed to **sad** stories?

How **happy** do happy stories usually make you feel?

How **sad** do sad stories usually make you feel?

When you choose to watch a show or movie, read a story or read a news item online, how often do you choose a **happy** one?

When you choose to watch a show or movie, read a story or read a news item online, how often do you choose a **sad** one?

How often do you think you benefit or learn from a **happy** story?

How often do you think you benefit or learn from a **sad** story?
